# Bis[μ-2-(2-pyridylmethyl­amino­meth­yl)phenolato]-κ^4^ *N*,*N*′,*O*:*O*;κ^4^ *O*:*N*,*N*′,*O*-bis­[(thio­cyanato-κ*N*)copper(II)]

**DOI:** 10.1107/S1600536809031742

**Published:** 2009-08-22

**Authors:** Gervas E. Assey, Yohannes Tesema, Teshome Yisgedu, Yilma Gultneh, Ray J. Butcher

**Affiliations:** a Department of Chemistry, Howard University, 525 College Street NW, Washington, DC 20059, USA

## Abstract

The centrosymmetric binuclear complex, [Cu_2_(C_13_H_13_N_2_O)_2_(NCS)_2_], formed *via* phenolate oxygen bridges, involves the Cu^II^ atoms in a distorted square-pyramidal coordination [τ = 0.197 (1)]. A Cu⋯Cu separation of 3.2281 (3) Å is observed. The in-plane Cu—O_phenolate_ distance [1.9342 (8) Å] is shorter than the axial distance [2.252 (8) Å]. The Cu—N_amine_ and Cu—N_py_ distances are similar [2.0095 (10) and 2.0192 (10) Å, respectively]. The Cu—N_thio­cyanate_ distance [1.9678 (11) Å] is in the range found for Cu—N distances in previously determined structures containing coordinated thio­cyanate anions. There is an inter­molecular hydrogen bond between the amine H atom and the S atom of a coordinated thio­cyanate anion.

## Related literature

For the chemical properties, ligand binding properties and the synthesis of related copper complexes: Kuzmic *et al.* (1992 ▶); Lim *et al.* (2006 ▶); Rogers & Wolf (2002 ▶); Sharma *et al.* (2008 ▶); Yisgedu (2001 ▶). For related structures, see: Assey *et al.* (2009 ▶); Biswas *et al.* (2005 ▶); Sarkar *et al.* (2006 ▶); Shakya *et al.* (2006 ▶); Wang & Li (2005 ▶); You & Zhu (2004 ▶, 2005 ▶); You (2005 ▶). For the τ parameter, see: Addison *et al.* (1984 ▶).
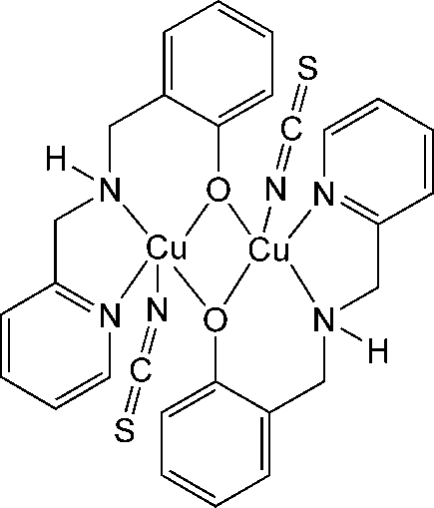


## Experimental

### 

#### Crystal data

[Cu_2_(C_13_H_13_N_2_O)_2_(NCS)_2_]
*M* *_r_* = 669.75
Monoclinic, 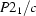

*a* = 7.4747 (1) Å
*b* = 16.9237 (3) Å
*c* = 11.0714 (2) Å
β = 91.1317 (18)°
*V* = 1400.25 (4) Å^3^
*Z* = 2
Mo *K*α radiation
μ = 1.71 mm^−1^
*T* = 200 K
0.44 × 0.37 × 0.28 mm

#### Data collection

Oxford Diffraction Gemini R diffractometer
Absorption correction: multi-scan (*CrysAlis RED*; Oxford Diffraction, 2008 ▶) *T*_min_ = 0.944, *T*_max_ = 1.000 (expected range = 0.586–0.620)
18586 measured reflections
7024 independent reflections
4611 reflections with *I* > 2σ(*I*)
*R*_int_ = 0.026

#### Refinement

*R*[*F* ^2^ > 2σ(*F* ^2^)] = 0.029
*wR*(*F* ^2^) = 0.071
*S* = 0.89
7024 reflections
181 parameters
H-atom parameters constrained
Δρ_max_ = 0.44 e Å^−3^
Δρ_min_ = −0.49 e Å^−3^

### 

Data collection: *CrysAlis CCD* (Oxford Diffraction, 2008 ▶); cell refinement: *CrysAlis RED* (Oxford Diffraction, 2008 ▶); data reduction: *CrysAlis RED*; program(s) used to solve structure: *SHELXS97* (Sheldrick, 2008 ▶); program(s) used to refine structure: *SHELXL97* (Sheldrick, 2008 ▶); molecular graphics: *SHELXTL* (Sheldrick, 2008 ▶); software used to prepare material for publication: *SHELXTL*.

## Supplementary Material

Crystal structure: contains datablocks I, global. DOI: 10.1107/S1600536809031742/kp2227sup1.cif

Structure factors: contains datablocks I. DOI: 10.1107/S1600536809031742/kp2227Isup2.hkl

Additional supplementary materials:  crystallographic information; 3D view; checkCIF report

## Figures and Tables

**Table:** 

Cu—O	1.9342 (8)
Cu—N	1.9678 (11)
Cu—N1	2.0095 (10)
Cu—N2	2.0192 (10)
Cu—O^i^	2.2526 (8)

**Table:** 

O—Cu—N	95.37 (4)
O—Cu—N1	92.66 (3)
N—Cu—N1	153.23 (5)
O—Cu—N2	165.03 (4)
N—Cu—N2	97.14 (4)
N1—Cu—N2	79.76 (4)
O—Cu—O^i^	79.39 (3)
N—Cu—O^i^	102.22 (4)
N1—Cu—O^i^	104.35 (3)
N2—Cu—O^i^	89.94 (3)

Symmetry code: (i) 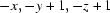.

**Table 2:** Hydrogen-bond geometry (Å, °)

*D*—H⋯*A*	*D*—H	H⋯*A*	*D*⋯*A*	*D*—H⋯*A*
N1—H1*A*⋯S^ii^	0.93	2.48	3.3866 (10)	164

Symmetry code: (ii) 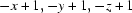.

**Fig. 1.**
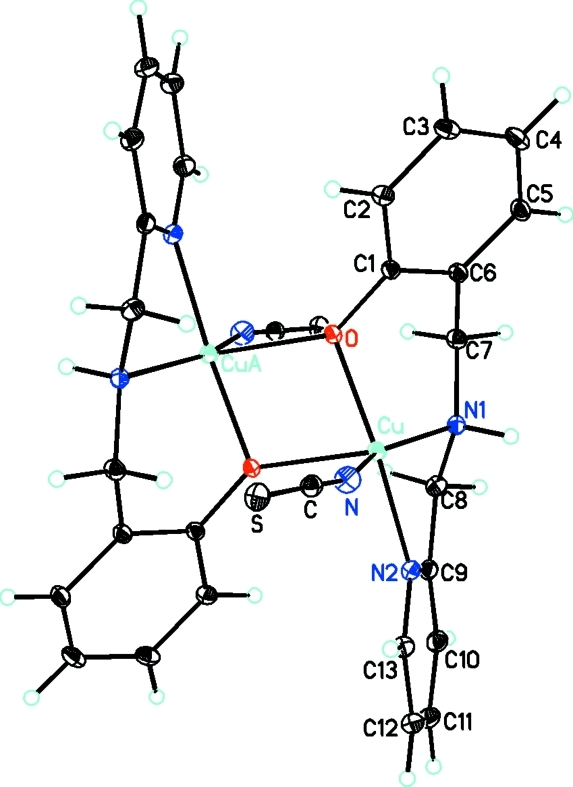
The molecular structure of the dinuclear complex I showing the atom numbering scheme and the 50% probability displacement ellipsoids. The symmetry code for generating the symmetry related dinuclear unit is -x, 1 - y, 1 - z.

**Fig. 2.**
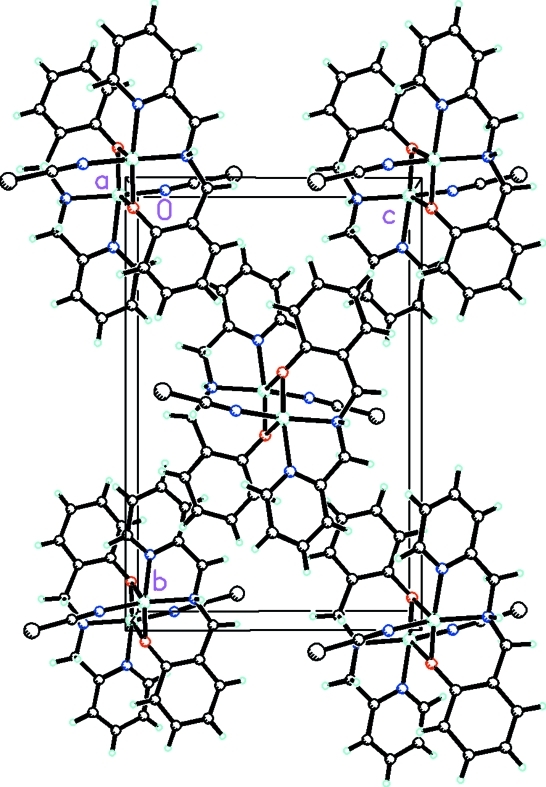
The molecular packing for I viewed down the a axis.

## supplementary crystallographic information

### Comment

The synthesis and structure determination of copper complexes containing potentially bridging or ambidentate ligands, such as the thiocyanate anion, is of wide interest. The different possible coordination modes of the thiocyanate anion can involve bridging or ambidentate functions. A major obstacle to a more comprehensive study of thiocyanate polynuclear complexes is the lack of rational synthetic procedures. One convenient synthetic strategy is use displacement of weakly coordinated anions such as perchlorate of trifluoromethanesulfonate. In this instance some control over resulting stereochemistry can be achieved.

The present complex was synthesized by displacement of weakly coordinated perchlorate anions from a structurally determined complex (Assey *et al.*, 2009) The crystal structure of the resulting complex, bis{µ-*O*-[2-((pyridin-2-ylmethylamino)methyl)phenolato]thiocyanatocopper(II)} has been determined. In this instance, two thiocyanate ligands were ligated to the copper(II) complex as a result of displacement of weakly coordinated perchlorate ligands. This method of ligand substitution has been used in the area of chemical sensors such as: competitive assay method involving the displacement of an indicator ligand (Sharma *et al.*, 2008), fluorescence displacement method involving determination of receptor-ligand binding constants (Kuzmic *et al.*, 1992), luminescence molecular sensing based on analyte coordination to the transition metal complexes (Rogers & Wolf, 2002), and the use of ligand substitution for fluorescence sensing in detection of NO in biological aqueous solution (Lim *et al.*, 2006).

The complex contains a reduced Schiff base, 2-{[(pyridine-2-ylmethyl)amino]methyl}phenolato and thiocyanate coordinated to copper. The phenolate O atoms bridge the two copper centres and the two thiocyanate anions are terminally coordinated to each copper through their N donors (Fig. 1 and Table 1). The resulting complex reveals a centre of inversion. The coordination geometry about each Cu^II^ is distorted square pyramidal (τ = 0.197 (1), Addison *et al.*, 1984) with the bridging O, N_amine_, N_pyridine_ and N_thiocyanate_ forming the base and a bridging O forming the apex. The Cu—O_base_ and Cu—O_apex_ distances are 1.9342 (8) and 2.2526 (8) Å respectively. The Cu—N_thiocyanate_ distance [1.9678 (11) Å] is in the range observed for other copper(II) complexes containing coordinated thiocyanate (Wang & Li, 2005; You & Zhu, 2004; You, 2005; You & Zhu, 2005). The bond distances to the central copper [Cu—N(1) 2.0095 (10) Å, Cu—N(2) 2.0292 (10) Å, Cu—O 1.9342 (8) Å] are comparable to those observed for related complexes (Shakya *et al.*, 2005) and (Biswas *et al.*, 2005).

### Experimental

The complex was synthesized in a three-step process. 1. The ligand (2-pyridylmethyl)(2-hydroxybenzyl)amine (*L*^1^H) was synthesized as described below (Yisgedu, 2001). To 5.4 g (50 mmol) of 2-(2-aminomethyl)pyridine in 10 ml of ethanol was added 6.1 g (50 mmol) of salicylaldehyde in 15 ml of ethanol which resulted in a deep yellow colour. The solution was left to stir for 30 m. A sodium borohydride solution (3 g NaBH_4_, 0.4 g NaOH, and 40.0 ml of H_2_O) were added dropwise. The solution changed to colourless and was left to stir for 1 h after adding all the NaBH_4_ solution. The volume of the solution was reduced to 20 ml after extracting three times with chloroform (3 *x* 40 ml). The extracts were combined and dried in anhydrous Na_2_SO_4_ overnight. The Na_2_SO_4_ was filtered and the filtrate concentrated to give a colourless oil (9.3 g, 87%).

2. Copper(II) perchlorate precursor complex was synthesized as follows (Yisgedu, 2001): 1.64 g (7.7 mmol) of *L*^1^H was mixed with 2.86 g (7.7 mmol) of Cu(ClO_4_)_2_.6H_2_O in 25 ml MeOH and 1.75 ml NaOCH_3_. The solution mixture was stirred overnight and a green filtrate of precipitate (2.7 g) was obtained. This was washed with EtOH/MeOH (2:1), then crystallized from CH_3_CN/CH_3_NO_2_.

3. Cu(II) thiocyanate complex was synthesized as follows: 0.1 mmol (0.0835 g) of the dinuclear Cu^II^ perchlorate complex was dissolved in 15 ml s of EtOH and reacted with 0.25 mmol (0.0203 g) of NaSCN dissolved in 10 ml s EtOH. The mixture was stirred for 18.5 h. A mixture of solids and supernatant were obtained at the end of the reaction. The supernatant was decanted and the solids were re-dissolved in 7.5 ml of DMF, filtered and layered with diethyl ether and left to crystallize. Dark-green crystals suitable for X-ray diffraction were obtained and used for X-ray structure determination.

### Refinement

H atoms were placed in geometrically idealized positions and constrained to ride on their parent atoms with C—H distances of 0.95 and 0.99 Å *U*_iso_(H) = 1.2*U*_eq_(C). The H atom attached to N was idealized with an N–H distance of 0.93 Å.

### Crystal data

**Table:**

[Cu_2_(C_13_H_13_N_2_O)_2_(NCS)_2_]	*F*(000) = 684
*M**_r_* = 669.75	*D*_x_ = 1.588 Mg m^−^^3^
Monoclinic, *P*2_1_/*c*	Mo *K*α radiation, λ = 0.71073 Å
*a* = 7.4747 (1) Å	Cell parameters from 7409 reflections
*b* = 16.9237 (3) Å	θ = 4.5–37.4°
*c* = 11.0714 (2) Å	µ = 1.71 mm^−^^1^
β = 91.1317 (18)°	*T* = 200 K
*V* = 1400.25 (4) Å^3^	Chunk, dark green
*Z* = 2	0.44 × 0.37 × 0.28 mm

### Data collection

**Table:**

Oxford Diffraction Gemini R diffractometer	7024 independent reflections
Radiation source: fine-focus sealed tube	4611 reflections with *I* > 2σ(*I*)
graphite	*R*_int_ = 0.026
Detector resolution: 10.5081 pixels mm^-1^	θ_max_ = 37.5°, θ_min_ = 4.5°
φ and ω scans	*h* = −12→12
Absorption correction: multi-scan (*CrysAlis RED*; Oxford Diffraction, 2008)	*k* = −28→25
*T*_min_ = 0.944, *T*_max_ = 1.000	*l* = −18→12
18586 measured reflections	

### Refinement

**Table:**

Refinement on *F*^2^	Primary atom site location: structure-invariant direct methods
Least-squares matrix: full	Secondary atom site location: difference Fourier map
*R*[*F*^2^ > 2σ(*F*^2^)] = 0.029	Hydrogen site location: inferred from neighbouring sites
*wR*(*F*^2^) = 0.071	H-atom parameters constrained
*S* = 0.89	*w* = 1/[σ^2^(*F*_o_^2^) + (0.037*P*)^2^] where *P* = (*F*_o_^2^ + 2*F*_c_^2^)/3
7024 reflections	(Δ/σ)_max_ = 0.004
181 parameters	Δρ_max_ = 0.44 e Å^−^^3^
0 restraints	Δρ_min_ = −0.49 e Å^−^^3^

### Special details

**Table:**

Experimental. CrysAlis RED, Oxford Diffraction Ltd., Version 1.171.32.15 (release 10-01-2008 CrysAlis171 .NET) (compiled Jan 10 2008,16:37:18) Empirical absorption correction using spherical harmonics, implemented in SCALE3 ABSPACK scaling algorithm.
Geometry. All e.s.d.'s (except the e.s.d. in the dihedral angle between two l.s. planes) are estimated using the full covariance matrix. The cell e.s.d.'s are taken into account individually in the estimation of e.s.d.'s in distances, angles and torsion angles; correlations between e.s.d.'s in cell parameters are only used when they are defined by crystal symmetry. An approximate (isotropic) treatment of cell e.s.d.'s is used for estimating e.s.d.'s involving l.s. planes.
Refinement. Refinement of *F*^2^ against ALL reflections. The weighted *R*-factor *wR* and goodness of fit *S* are based on *F*^2^, conventional *R*-factors *R* are based on *F*, with *F* set to zero for negative *F*^2^. The threshold expression of *F*^2^ > σ(*F*^2^) is used only for calculating *R*-factors(gt) *etc*. and is not relevant to the choice of reflections for refinement. *R*-factors based on *F*^2^ are statistically about twice as large as those based on *F*, and *R*- factors based on ALL data will be even larger.

### Fractional atomic coordinates and isotropic or equivalent isotropic displacement parameters (Å^2^)

**Table:**

	*x*	*y*	*z*	*U*_iso_*/*U*_eq_	
Cu	0.200074 (18)	0.530744 (8)	0.531057 (12)	0.01989 (4)	
S	0.40414 (4)	0.472939 (18)	0.13944 (3)	0.02856 (7)	
O	0.07568 (10)	0.43052 (4)	0.52958 (7)	0.02117 (15)	
N	0.31981 (15)	0.51404 (7)	0.37642 (10)	0.0315 (2)	
N1	0.19612 (12)	0.53813 (5)	0.71214 (9)	0.02082 (18)	
H1A	0.3146	0.5407	0.7390	0.025*	
N2	0.27182 (13)	0.64484 (6)	0.55436 (9)	0.02351 (19)	
C	0.35464 (15)	0.49643 (7)	0.27835 (11)	0.0232 (2)	
C1	0.13193 (14)	0.37486 (6)	0.60789 (10)	0.0201 (2)	
C2	0.15668 (15)	0.29704 (7)	0.56836 (11)	0.0243 (2)	
H2A	0.1256	0.2832	0.4875	0.029*	
C3	0.22630 (16)	0.23993 (7)	0.64630 (12)	0.0286 (3)	
H3A	0.2415	0.1873	0.6185	0.034*	
C4	0.27368 (17)	0.25921 (8)	0.76442 (12)	0.0314 (3)	
H4A	0.3271	0.2208	0.8163	0.038*	
C5	0.24208 (16)	0.33520 (7)	0.80592 (11)	0.0271 (2)	
H5A	0.2722	0.3483	0.8873	0.033*	
C6	0.16704 (15)	0.39265 (7)	0.73042 (10)	0.0216 (2)	
C7	0.11123 (16)	0.47201 (6)	0.77868 (11)	0.0238 (2)	
H7A	−0.0205	0.4770	0.7716	0.029*	
H7B	0.1453	0.4756	0.8654	0.029*	
C8	0.11526 (16)	0.61569 (7)	0.73720 (11)	0.0253 (2)	
H8A	0.1373	0.6305	0.8227	0.030*	
H8B	−0.0156	0.6136	0.7220	0.030*	
C9	0.19939 (15)	0.67555 (6)	0.65499 (11)	0.0232 (2)	
C10	0.20102 (17)	0.75594 (7)	0.67794 (12)	0.0293 (3)	
H10A	0.1478	0.7766	0.7485	0.035*	
C11	0.28194 (18)	0.80584 (7)	0.59583 (14)	0.0351 (3)	
H11A	0.2836	0.8613	0.6091	0.042*	
C12	0.35985 (17)	0.77426 (7)	0.49476 (13)	0.0330 (3)	
H12A	0.4180	0.8075	0.4386	0.040*	
C13	0.35215 (17)	0.69330 (7)	0.47618 (12)	0.0287 (2)	
H13A	0.4051	0.6715	0.4063	0.034*	

### Atomic displacement parameters (Å^2^)

**Table:**

	*U*^11^	*U*^22^	*U*^33^	*U*^12^	*U*^13^	*U*^23^
Cu	0.02292 (7)	0.01935 (6)	0.01741 (7)	−0.00059 (5)	0.00062 (5)	−0.00060 (5)
S	0.03068 (15)	0.03244 (15)	0.02262 (15)	0.00118 (12)	0.00194 (11)	−0.00631 (12)
O	0.0239 (4)	0.0196 (3)	0.0198 (4)	0.0005 (3)	−0.0051 (3)	0.0021 (3)
N	0.0326 (6)	0.0367 (6)	0.0256 (5)	−0.0008 (4)	0.0074 (4)	−0.0037 (4)
N1	0.0204 (4)	0.0215 (4)	0.0205 (5)	0.0007 (3)	−0.0009 (3)	−0.0009 (3)
N2	0.0214 (4)	0.0230 (4)	0.0260 (5)	−0.0020 (3)	−0.0025 (4)	0.0003 (4)
C	0.0199 (5)	0.0226 (5)	0.0271 (6)	0.0003 (4)	0.0010 (4)	0.0003 (4)
C1	0.0180 (4)	0.0210 (5)	0.0215 (5)	0.0000 (4)	0.0004 (4)	0.0033 (4)
C2	0.0268 (5)	0.0218 (5)	0.0243 (6)	0.0014 (4)	0.0018 (4)	0.0008 (4)
C3	0.0298 (6)	0.0207 (5)	0.0356 (7)	0.0043 (4)	0.0055 (5)	0.0048 (5)
C4	0.0310 (6)	0.0296 (6)	0.0336 (7)	0.0067 (5)	0.0014 (5)	0.0138 (5)
C5	0.0282 (6)	0.0311 (6)	0.0219 (6)	0.0002 (5)	−0.0026 (4)	0.0068 (5)
C6	0.0216 (5)	0.0229 (5)	0.0204 (5)	−0.0007 (4)	−0.0002 (4)	0.0041 (4)
C7	0.0284 (5)	0.0253 (5)	0.0176 (5)	−0.0001 (4)	0.0024 (4)	0.0015 (4)
C8	0.0280 (6)	0.0230 (5)	0.0249 (6)	0.0006 (4)	0.0024 (4)	−0.0056 (4)
C9	0.0223 (5)	0.0223 (5)	0.0249 (6)	0.0002 (4)	−0.0045 (4)	−0.0026 (4)
C10	0.0313 (6)	0.0236 (5)	0.0329 (7)	0.0002 (5)	−0.0042 (5)	−0.0055 (5)
C11	0.0353 (7)	0.0211 (5)	0.0484 (9)	−0.0039 (5)	−0.0095 (6)	−0.0004 (5)
C12	0.0302 (6)	0.0279 (6)	0.0405 (8)	−0.0065 (5)	−0.0043 (5)	0.0071 (5)
C13	0.0270 (5)	0.0294 (6)	0.0297 (7)	−0.0040 (5)	0.0000 (5)	0.0036 (5)

### Geometric parameters (Å, °)

**Table:**

Cu—O	1.9342 (8)	C3—H3A	0.9500
Cu—N	1.9678 (11)	C4—C5	1.3874 (18)
Cu—N1	2.0095 (10)	C4—H4A	0.9500
Cu—N2	2.0192 (10)	C5—C6	1.3929 (16)
Cu—O^i^	2.2526 (8)	C5—H5A	0.9500
S—C	1.6378 (12)	C6—C7	1.5073 (16)
O—C1	1.3421 (13)	C7—H7A	0.9900
O—Cu^i^	2.2526 (8)	C7—H7B	0.9900
N—C	1.1605 (16)	C8—C9	1.5076 (16)
N1—C8	1.4737 (14)	C8—H8A	0.9900
N1—C7	1.4886 (14)	C8—H8B	0.9900
N1—H1A	0.9300	C9—C10	1.3840 (16)
N2—C13	1.3427 (15)	C10—C11	1.3882 (19)
N2—C9	1.3521 (15)	C10—H10A	0.9500
C1—C2	1.4012 (15)	C11—C12	1.379 (2)
C1—C6	1.4092 (16)	C11—H11A	0.9500
C2—C3	1.3900 (17)	C12—C13	1.3865 (18)
C2—H2A	0.9500	C12—H12A	0.9500
C3—C4	1.387 (2)	C13—H13A	0.9500
			
O—Cu—N	95.37 (4)	C5—C4—H4A	120.4
O—Cu—N1	92.66 (3)	C4—C5—C6	121.16 (12)
N—Cu—N1	153.23 (5)	C4—C5—H5A	119.4
O—Cu—N2	165.03 (4)	C6—C5—H5A	119.4
N—Cu—N2	97.14 (4)	C5—C6—C1	119.59 (11)
N1—Cu—N2	79.76 (4)	C5—C6—C7	121.35 (11)
O—Cu—O^i^	79.39 (3)	C1—C6—C7	118.94 (10)
N—Cu—O^i^	102.22 (4)	N1—C7—C6	111.76 (9)
N1—Cu—O^i^	104.35 (3)	N1—C7—H7A	109.3
N2—Cu—O^i^	89.94 (3)	C6—C7—H7A	109.3
C1—O—Cu	117.75 (7)	N1—C7—H7B	109.3
C1—O—Cu^i^	132.06 (7)	C6—C7—H7B	109.3
Cu—O—Cu^i^	100.61 (3)	H7A—C7—H7B	107.9
C—N—Cu	164.84 (11)	N1—C8—C9	107.90 (9)
C8—N1—C7	113.33 (9)	N1—C8—H8A	110.1
C8—N1—Cu	104.92 (7)	C9—C8—H8A	110.1
C7—N1—Cu	117.50 (7)	N1—C8—H8B	110.1
C8—N1—H1A	106.8	C9—C8—H8B	110.1
C7—N1—H1A	106.8	H8A—C8—H8B	108.4
Cu—N1—H1A	106.8	N2—C9—C10	121.79 (11)
C13—N2—C9	119.20 (10)	N2—C9—C8	114.69 (9)
C13—N2—Cu	128.51 (9)	C10—C9—C8	123.50 (11)
C9—N2—Cu	111.30 (7)	C9—C10—C11	118.70 (12)
N—C—S	179.16 (12)	C9—C10—H10A	120.7
O—C1—C2	119.97 (10)	C11—C10—H10A	120.7
O—C1—C6	121.46 (10)	C12—C11—C10	119.47 (12)
C2—C1—C6	118.57 (10)	C12—C11—H11A	120.3
C3—C2—C1	120.65 (12)	C10—C11—H11A	120.3
C3—C2—H2A	119.7	C11—C12—C13	119.10 (12)
C1—C2—H2A	119.7	C11—C12—H12A	120.5
C4—C3—C2	120.48 (12)	C13—C12—H12A	120.5
C4—C3—H3A	119.8	N2—C13—C12	121.71 (12)
C2—C3—H3A	119.8	N2—C13—H13A	119.1
C3—C4—C5	119.23 (11)	C12—C13—H13A	119.1
C3—C4—H4A	120.4		
			
N—Cu—O—C1	108.23 (8)	Cu^i^—O—C1—C6	−92.44 (11)
N1—Cu—O—C1	−46.20 (8)	O—C1—C2—C3	175.63 (10)
N2—Cu—O—C1	−105.16 (15)	C6—C1—C2—C3	−4.46 (16)
O^i^—Cu—O—C1	−150.31 (9)	C1—C2—C3—C4	−0.47 (18)
N—Cu—O—Cu^i^	−101.46 (4)	C2—C3—C4—C5	3.37 (18)
N1—Cu—O—Cu^i^	104.11 (4)	C3—C4—C5—C6	−1.27 (19)
N2—Cu—O—Cu^i^	45.15 (15)	C4—C5—C6—C1	−3.70 (18)
O^i^—Cu—O—Cu^i^	0.0	C4—C5—C6—C7	172.27 (11)
O—Cu—N—C	37.5 (4)	O—C1—C6—C5	−173.61 (10)
N1—Cu—N—C	144.3 (4)	C2—C1—C6—C5	6.48 (16)
N2—Cu—N—C	−134.3 (4)	O—C1—C6—C7	10.32 (15)
O^i^—Cu—N—C	−42.8 (4)	C2—C1—C6—C7	−169.59 (10)
O—Cu—N1—C8	−126.33 (7)	C8—N1—C7—C6	166.99 (10)
N—Cu—N1—C8	126.22 (10)	Cu—N1—C7—C6	44.27 (12)
N2—Cu—N1—C8	40.68 (7)	C5—C6—C7—N1	125.30 (11)
O^i^—Cu—N1—C8	−46.61 (7)	C1—C6—C7—N1	−58.71 (14)
O—Cu—N1—C7	0.59 (8)	C7—N1—C8—C9	−174.09 (9)
N—Cu—N1—C7	−106.86 (11)	Cu—N1—C8—C9	−44.65 (10)
N2—Cu—N1—C7	167.59 (8)	C13—N2—C9—C10	1.96 (17)
O^i^—Cu—N1—C7	80.31 (8)	Cu—N2—C9—C10	−167.62 (9)
O—Cu—N2—C13	−137.76 (14)	C13—N2—C9—C8	−179.11 (10)
N—Cu—N2—C13	8.72 (11)	Cu—N2—C9—C8	11.31 (12)
N1—Cu—N2—C13	161.81 (11)	N1—C8—C9—N2	22.59 (14)
O^i^—Cu—N2—C13	−93.59 (10)	N1—C8—C9—C10	−158.50 (11)
O—Cu—N2—C9	30.60 (19)	N2—C9—C10—C11	−1.00 (19)
N—Cu—N2—C9	177.08 (8)	C8—C9—C10—C11	−179.83 (12)
N1—Cu—N2—C9	−29.83 (8)	C9—C10—C11—C12	−0.7 (2)
O^i^—Cu—N2—C9	74.77 (8)	C10—C11—C12—C13	1.4 (2)
Cu—N—C—S	117 (8)	C9—N2—C13—C12	−1.24 (18)
Cu—O—C1—C2	−133.51 (9)	Cu—N2—C13—C12	166.32 (10)
Cu^i^—O—C1—C2	87.46 (12)	C11—C12—C13—N2	−0.4 (2)
Cu—O—C1—C6	46.58 (12)		

Symmetry codes: (i) −*x*, −*y*+1, −*z*+1.

### Hydrogen-bond geometry (Å, °)

**Table:**

*D*—H···*A*	*D*—H	H···*A*	*D*···*A*	*D*—H···*A*
N1—H1A···S^ii^	0.93	2.48	3.3866 (10)	164

Symmetry codes: (ii) −*x*+1, −*y*+1, −*z*+1.
